# CULTURE-RELATED TASK DIFFICULTIES AND NEGATIVE CONSEQUENCES FOR CAREGIVERS FROM DIVERSE COMMUNITIES

**DOI:** 10.1093/geroni/igz038.3461

**Published:** 2019-11-08

**Authors:** Rachel Schaffer, David Bass, Sara Powers, Jenna McDavid, Ocean Le

**Affiliations:** 1 Benjamin Rose Institute on Aging, Cleveland, Ohio, United States; 2 Diverse Elders Coalition, New York, New York, United States

## Abstract

The Diverse Elders Coalition, in partnership with its six member organizations and the Benjamin Rose Institute on Aging, completed a national survey of 840 family and friend caregivers from diverse racial, ethnic, and sexual orientation communities to understand their unique caregiving issues and challenges. Data from a subsample of 404 caregivers identifying as Hispanic/Latino, Asian, Southeast Asian or from multiple ethnicities were examined to determine the relationship between difficulties performing culture-related care tasks (i.e., assisting with immigration issues and language barriers) and a variety of caregiver outcomes. Regression analysis controlling for background and context characteristics showed caregivers experiencing more culture-related task difficulties had significantly higher levels of several negative caregiving consequences, including health strain (B=.19, p=.001), relationship strain (B=.17, p=.005), work strain, (B=.24, p=.000) isolation (B=.17, p=.002), and symptoms of depression (B=.29, p=.000). Moreover, higher levels of strain and depression among caregivers who reported high levels of culture-related task difficulties ranged from 26%-54%. More difficulties with culture-related tasks also were significantly related to dissatisfaction with support resources, including lower ratings of the quality of care receivers’ healthcare (B=-.20, p=.001), and lower satisfaction with support they and their care receivers received from family and friends (B=-.17, p=.006 and B=-.16, p=.011, respectively). Results suggest caregivers from diverse communities struggling with culture-related tasks experience more negative consequences of caregiving and less helpful social support. Service providers working with caregivers from diverse communities should assess for culture-related task difficulties, recognizing these problems may be a window into a variety of adverse caregiving effects.

